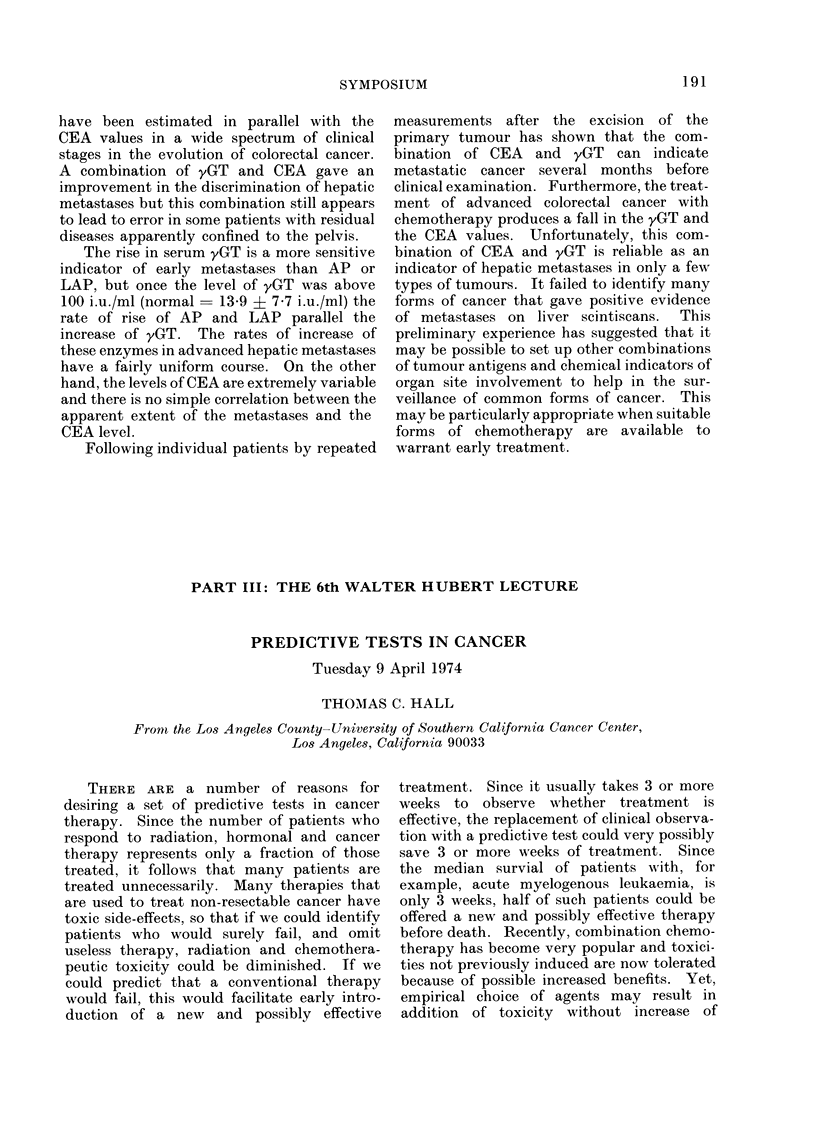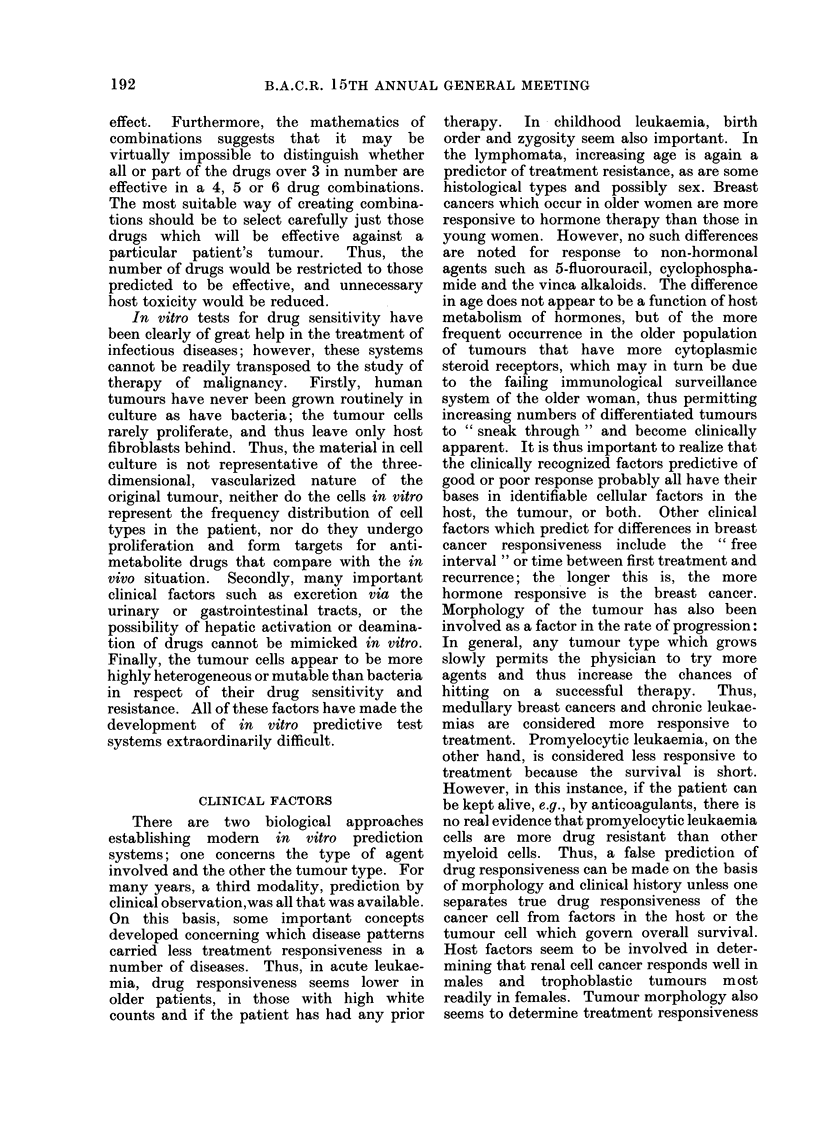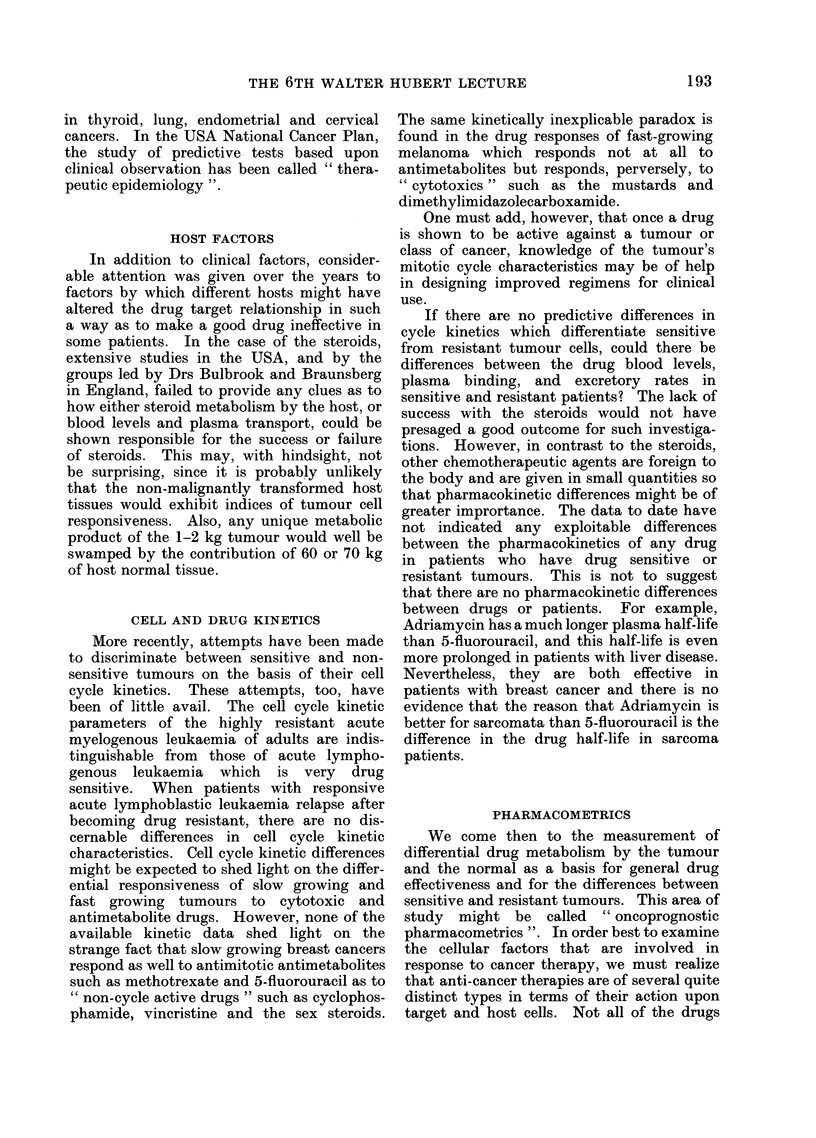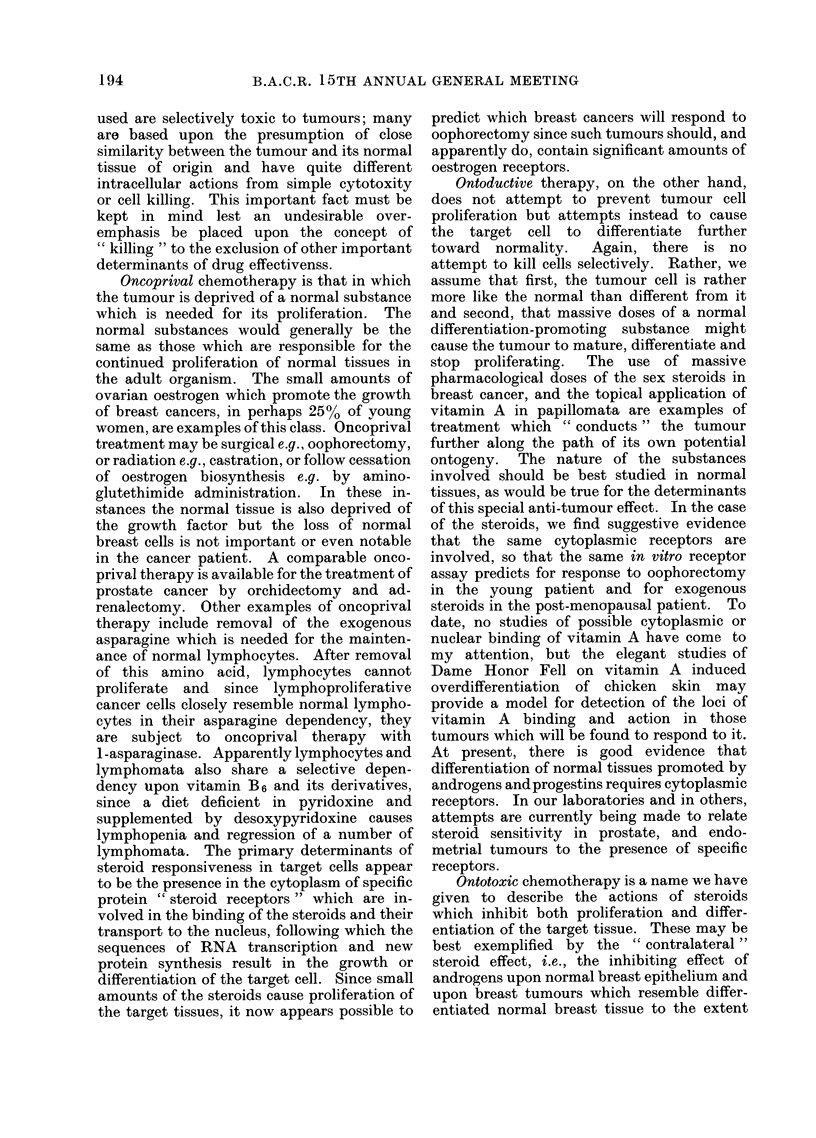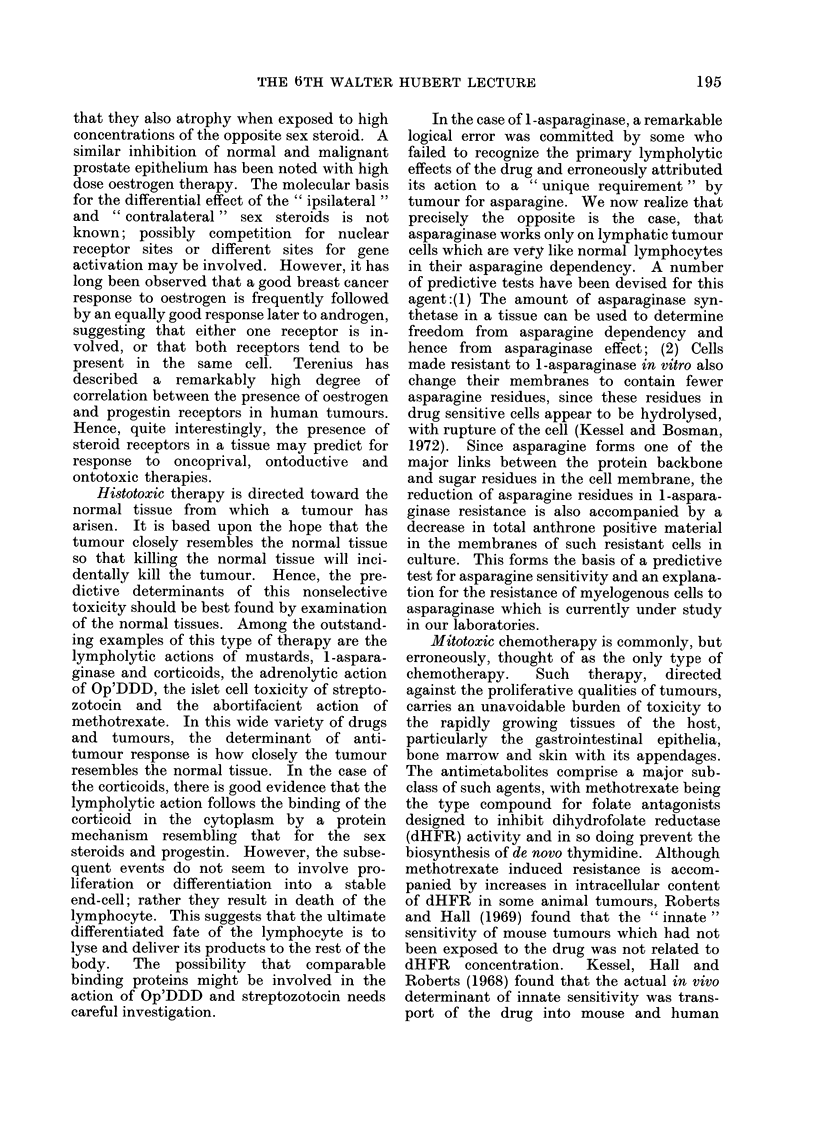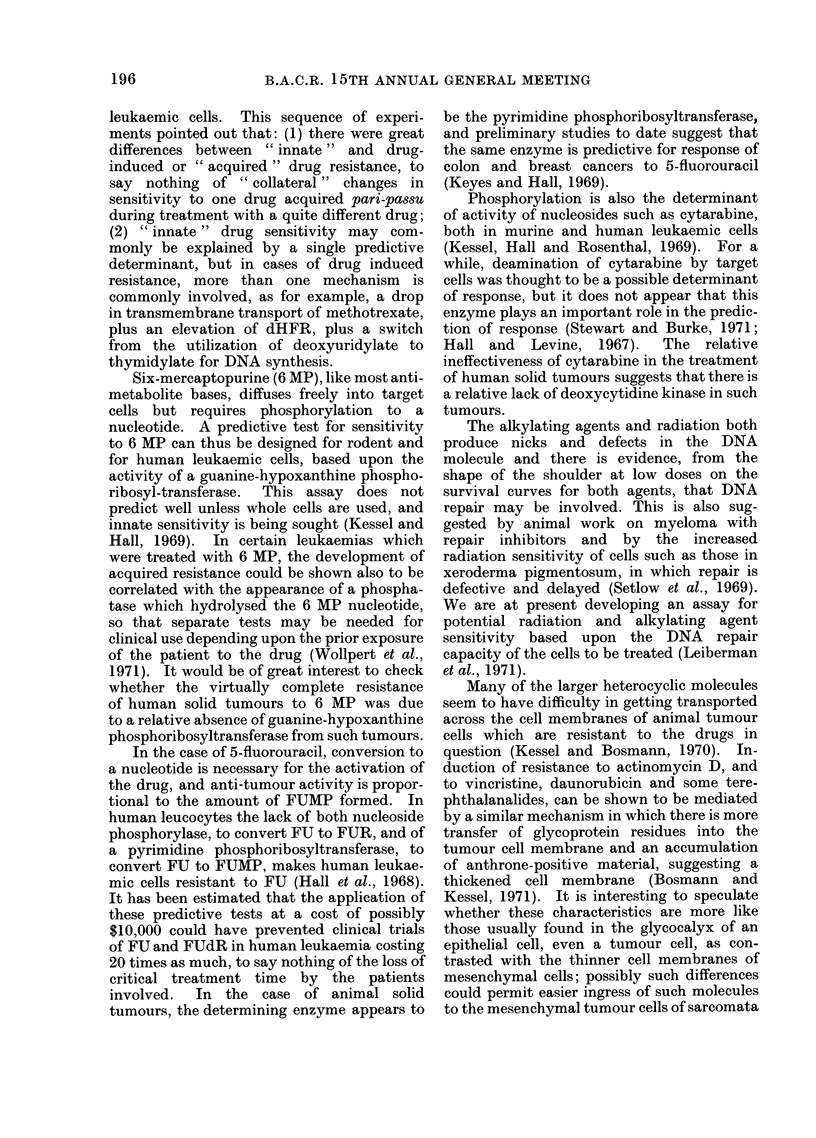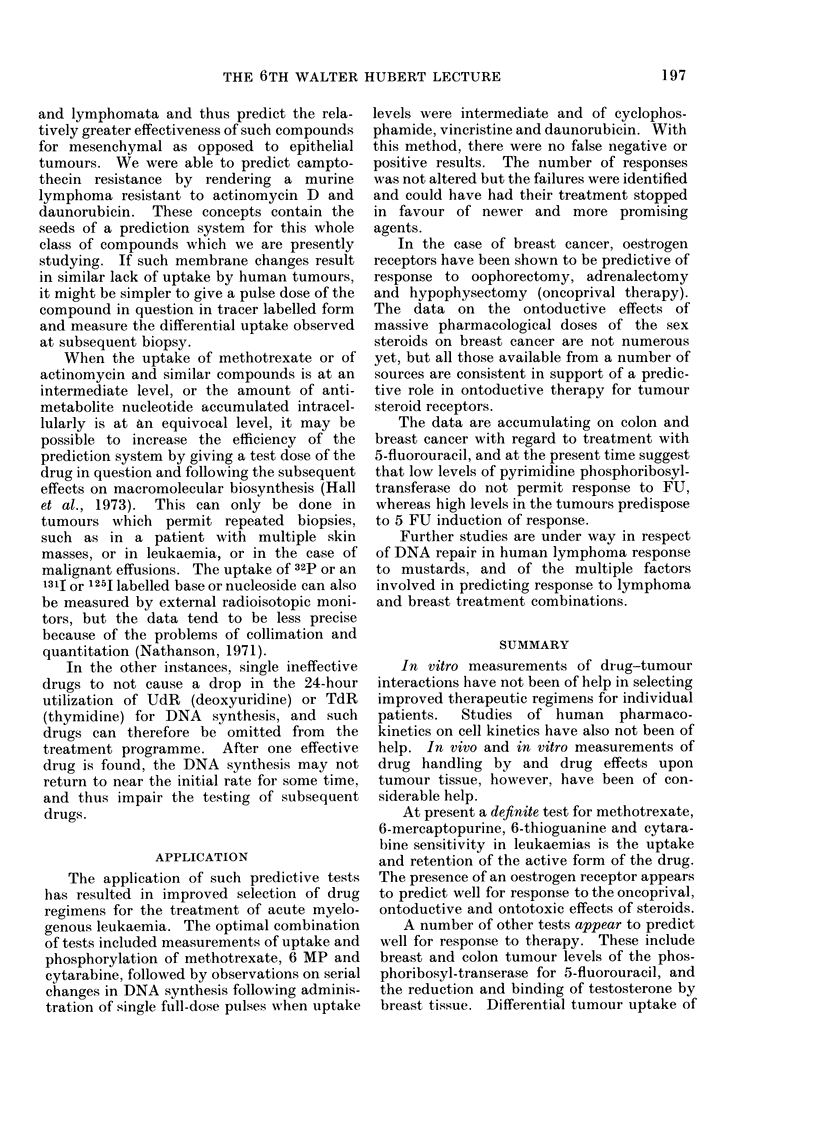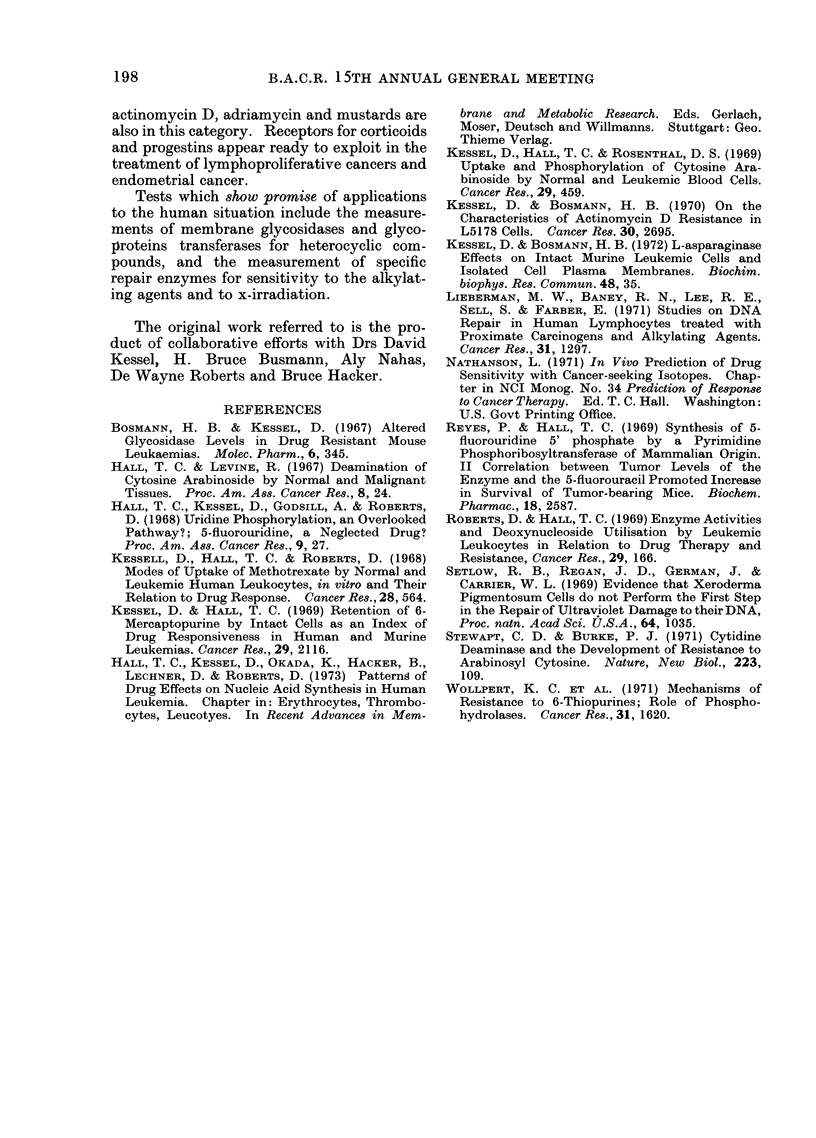# Predictive tests in cancer. Tuesday 9 April 1974.

**DOI:** 10.1038/bjc.1974.181

**Published:** 1974-08

**Authors:** T. C. Hall


### PART III: THE 6th WALTER HUBERT LECTURE

### PREDICTIVE TESTS IN CANCER

Tuesday 9 April 1974

THOMAS C. HALL

*From the Los Angeles County–University of Southern California Cancer Center, Los Angeles, California* 90033

THERE ARE a number of reasons for desiring a set of predictive tests in cancer therapy. Since the number of patients who respond to radiation, hormonal and cancer therapy represents only a fraction of those treated, it follows that many patients are treated unnecessarily. Many therapies that are used to treat non-resectable cancer have toxic side-effects, so that if we could identify patients who would surely fail, and omit useless therapy, radiation and chemotherapeutic toxicity could be diminished. If we could predict that a conventional therapy would fail, this would facilitate early introduction of a new and possibly effective treatment. Since it usually takes 3 or more weeks to observe whether treatment is effective, the replacement of clinical observation with a predictive test could very possibly save 3 or more weeks of treatment. Since the median survial of patients with, for example, acute myelogenous leukaemia, is only 3 weeks, half of such patients could be offered a new and possibly effective therapy before death. Recently, combination chemotherapy has become very popular and toxicities not previously induced are now tolerated because of possible increased benefits. Yet, empirical choice of agents may result in addition of toxicity without increase of effect. Furthermore, the mathematics of combinations suggests that it may be virtually impossible to distinguish whether all or part of the drugs over 3 in number are effective in a 4, 5 or 6 drug combinations. The most suitable way of creating combinations should be to select carefully just those drugs which will be effective against a particular patient's tumour. Thus, the number of drugs would be restricted to those predicted to be effective, and unnecessary host toxicity would be reduced.

*In vitro* tests for drug sensitivity have been clearly of great help in the treatment of infectious diseases; however, these systems cannot be readily transposed to the study of therapy of malignancy. Firstly, human tumours have never been grown routinely in culture as have bacteria; the tumour cells rarely proliferate, and thus leave only host fibroblasts behind. Thus, the material in cell culture is not representative of the three-dimensional, vascularized nature of the original tumour, neither do the cells *in vitro* represent the frequency distribution of cell types in the patient, nor do they undergo proliferation and form targets for anti-metabolite drugs that compare with the *in vivo* situation. Secondly, many important clinical factors such as excretion *via* the urinary or gastrointestinal tracts, or the possibility of hepatic activation or deamination of drugs cannot be mimicked *in vitro*. Finally, the tumour cells appear to be more highly heterogeneous or mutable than bacteria in respect of their drug sensitivity and resistance. All of these factors have made the development of *in vitro* predictive test systems extraordinarily difficult.

### CLINICAL FACTORS

There are two biological approaches establishing modern *in vitro* prediction systems; one concerns the type of agent involved and the other the tumour type. For many years, a third modality, prediction by clinical observation, was all that was available. On this basis, some important concepts developed concerning which disease patterns carried less treatment responsiveness in a number of diseases. Thus, in acute leukaemia, drug responsiveness seems lower in older patients, in those with high white counts and if the patient has had any prior therapy. In childhood leukaemia, birth order and zygosity seem also important. In the lymphomata, increasing age is again a predictor of treatment resistance, as are some histological types and possibly sex. Breast cancers which occur in older women are more responsive to hormone therapy than those in young women. However, no such differences are noted for response to non-hormonal agents such as 5-fluorouracil, cyclophosphamide and the vinca alkaloids. The difference in age does not appear to be a function of host metabolism of hormones, but of the more frequent occurrence in the older population of tumours that have more cytoplasmic steroid receptors, which may in turn be due to the failing immunological surveillance system of the older woman, thus permitting increasing numbers of differentiated tumours to " sneak through " and become clinically apparent. It is thus important to realize that the clinically recognized factors predictive of good or poor response probably all have their bases in identifiable cellular factors in the host, the tumour, or both. Other clinical factors which predict for differences in breast cancer responsiveness include the " free interval " or time between first treatment and recurrence; the longer this is, the more hormone responsive is the breast cancer. Morphology of the tumour has also been involved as a factor in the rate of progression: In general, any tumour type which grows slowly permits the physician to try more agents and thus increase the chances of hitting on a successful therapy. Thus, medullary breast cancers and chronic leukaemias are considered more responsive to treatment. Promyelocytic leukaemia, on the other hand, is considered less responsive to treatment because the survival is short. However, in this instance, if the patient can be kept alive, *e.g.*, by anticoagulants, there is no real evidence that promyelocytic leukaemia cells are more drug resistant than other myeloid cells. Thus, a false prediction of drug responsiveness can be made on the basis of morphology and clinical history unless one separates true drug responsiveness of the cancer cell from factors in the host or the tumour cell which govern overall survival. Host factors seem to be involved in determining that renal cell cancer responds well in males and trophoblastic tumours most readily in females. Tumour morphology also seems to determine treatment responsiveness in thyroid, lung, endometrial and cervical cancers. In the USA National Cancer Plan, the study of predictive tests based upon clinical observation has been called " therapeutic epidemiology ".

### HOST FACTORS

In addition to clinical factors, considerable attention was given over the years to factors by which different hosts might have altered the drug target relationship in such a way as to make a good drug ineffective in some patients. In the case of the steroids, extensive studies in the USA, and by the groups led by Drs Bulbrook and Braunsberg in England, failed to provide any clues as to how either steroid metabolism by the host, or blood levels and plasma transport, could be shown responsible for the success or failure of steroids. This may, with hindsight, not be surprising, since it is probably unlikely that the non-malignantly transformed host tissues would exhibit indices of tumour cell responsiveness. Also, any unique metabolic product of the 1–2 kg tumour would well be swamped by the contribution of 60 or 70 kg of host normal tissue.

### CELL AND DRUG KINETICS

More recently, attempts have been made to discriminate between sensitive and non-sensitive tumours on the basis of their cell cycle kinetics. These attempts, too, have been of little avail. The cell cycle kinetic parameters of the highly resistant acute myelogenous leukaemia of adults are indistinguishable from those of acute lymphogenous leukaemia which is very drug sensitive. When patients with responsive acute lymphoblastic leukaemia relapse after becoming drug resistant, there are no discernable differences in cell cycle kinetic characteristics. Cell cycle kinetic differences might be expected to shed light on the differential responsiveness of slow growing and fast growing tumours to cytotoxic and antimetabolite drugs. However, none of the available kinetic data shed light on the strange fact that slow growing breast cancers respond as well to antimitotic antimetabolites such as methotrexate and 5-fluorouracil as to " non-cycle active drugs " such as cyclophosphamide, vincristine and the sex steroids. The same kinetically inexplicable paradox is found in the drug responses of fast-growing melanoma which responds not at all to antimetabolites but responds, perversely, to " cytotoxics " such as the mustards and dimethylimidazolecarboxamide.

One must add, however, that once a drug is shown to be active against a tumour or class of cancer, knowledge of the tumour's mitotic cycle characteristics may be of help in designing improved regimens for clinical use.

If there are no predictive differences in cycle kinetics which differentiate sensitive from resistant tumour cells, could there be differences between the drug blood levels, plasma binding, and excretory rates in sensitive and resistant patients? The lack of success with the steroids would not have presaged a good outcome for such investigations. However, in contrast to the steroids, other chemotherapeutic agents are foreign to the body and are given in small quantities so that pharmacokinetic differences might be of greater imprortance. The data to date have not indicated any exploitable differences between the pharmacokinetics of any drug in patients who have drug sensitive or resistant tumours. This is not to suggest that there are no pharmacokinetic differences between drugs or patients. For example, Adriamycin has a much longer plasma half-life than 5-fluorouracil, and this half-life is even more prolonged in patients with liver disease. Nevertheless, they are both effective in patients with breast cancer and there is no evidence that the reason that Adriamycin is better for sarcomata than 5-fluorouracil is the difference in the drug half-life in sarcoma patients.

### PHARMACOMETRICS

We come then to the measurement of differential drug metabolism by the tumour and the normal as a basis for general drug effectiveness and for the differences between sensitive and resistant tumours. This area of study might be called " oncoprognostic pharmacometrics ". In order best to examine the cellular factors that are involved in response to cancer therapy, we must realize that anti-cancer therapies are of several quite distinct types in terms of their action upon target and host cells. Not all of the drugs used are selectively toxic to tumours; many are based upon the presumption of close similarity between the tumour and its normal tissue of origin and have quite different intracellular actions from simple cytotoxity or cell killing. This important fact must be kept in mind lest an undesirable over-emphasis be placed upon the concept of " killing " to the exclusion of other important determinants of drug effectivenss.

*Oncoprival* chemotherapy is that in which the tumour is deprived of a normal substance which is needed for its proliferation. The normal substances would generally be the same as those which are responsible for the continued proliferation of normal tissues in the adult organism. The small amounts of ovarian oestrogen which promote the growth of breast cancers, in perhaps 25% of young women, are examples of this class. Oncoprival treatment may be surgical *e.g.*, oophorectomy, or radiation *e.g.*, castration, or follow cessation of oestrogen biosynthesis *e.g.* by amino-glutethimide administration. In these instances the normal tissue is also deprived of the growth factor but the loss of normal breast cells is not important or even notable in the cancer patient. A comparable oncoprival therapy is available for the treatment of prostate cancer by orchidectomy and adrenalectomy. Other examples of oncoprival therapy include removal of the exogenous asparagine which is needed for the maintenance of normal lymphocytes. After removal of this amino acid, lymphocytes cannot proliferate and since lymphoproliferative cancer cells closely resemble normal lymphocytes in their asparagine dependency, they are subject to oncoprival therapy with l-asparaginase. Apparently lymphocytes and lymphomata also share a selective dependency upon vitamin $B_6$ and its derivatives, since a diet deficient in pyridoxine and supplemented by desoxypyridoxine causes lymphopenia and regression of a number of lymphomata. The primary determinants of steroid responsiveness in target cells appear to be the presence in the cytoplasm of specific protein " steroid receptors " which are involved in the binding of the steroids and their transport to the nucleus, following which the sequences of RNA transcription and new protein synthesis result in the growth or differentiation of the target cell. Since small amounts of the steroids cause proliferation of the target tissues, it now appears possible to predict which breast cancers will respond to oophorectomy since such tumours should, and apparently do, contain significant amounts of oestrogen receptors.

*Ontoductive* therapy, on the other hand, does not attempt to prevent tumour cell proliferation but attempts instead to cause the target cell to differentiate further toward normality. Again, there is no attempt to kill cells selectively. Rather, we assume that first, the tumour cell is rather more like the normal than different from it and second, that massive doses of a normal differentiation-promoting substance might cause the tumour to mature, differentiate and stop proliferating. The use of massive pharmacological doses of the sex steroids in breast cancer, and the topical application of vitamin A in papillomata are examples of treatment which " conducts " the tumour further along the path of its own potential ontogeny. The nature of the substances involved should be best studied in normal tissues, as would be true for the determinants of this special anti-tumour effect. In the case of the steroids, we find suggestive evidence that the same cytoplasmic receptors are involved, so that the same *in vitro* receptor assay predicts for response to oophorectomy in the young patient and for exogenous steroids in the post-menopausal patient. To date, no studies of possible cytoplasmic or nuclear binding of vitamin A have come to my attention, but the elegant studies of Dame Honor Fell on vitamin A induced overdifferentiation of chicken skin may provide a model for detection of the loci of vitamin A binding and action in those tumours which will be found to respond to it. At present, there is good evidence that differentiation of normal tissues promoted by androgens and progestins requires cytoplasmic receptors. In our laboratories and in others, attempts are currently being made to relate steroid sensitivity in prostate, and endometrial tumours to the presence of specific receptors.

*Ontotoxic* chemotherapy is a name we have given to describe the actions of steroids which inhibit both proliferation and differentiation of the target tissue. These may be best exemplified by the " contralateral " steroid effect, *i.e.*, the inhibiting effect of androgens upon normal breast epithelium and upon breast tumours which resemble differentiated normal breast tissue to the extent that they also atrophy when exposed to high concentrations of the opposite sex steroid. A similar inhibition of normal and malignant prostate epithelium has been noted with high dose oestrogen therapy. The molecular basis for the differential effect of the " ipsilateral " and " contralateral " sex steroids is not known; possibly competition for nuclear receptor sites or different sites for gene activation may be involved. However, it has long been observed that a good breast cancer response to oestrogen is frequently followed by an equally good response later to androgen, suggesting that either one receptor is involved, or that both receptors tend to be present in the same cell. Terenius has described a remarkably high degree of correlation between the presence of oestrogen and progestin receptors in human tumours. Hence, quite interestingly, the presence of steroid receptors in a tissue may predict for response to oncoprival, ontoductive and ontotoxic therapies.

*Histotoxic* therapy is directed toward the normal tissue from which a tumour has arisen. It is based upon the hope that the tumour closely resembles the normal tissue so that killing the normal tissue will incidentally kill the tumour. Hence, the predictive determinants of this nonselective toxicity should be best found by examination of the normal tissues. Among the outstanding examples of this type of therapy are the lympholytic actions of mustards, l-asparaginase and corticoids, the adrenolytic action of Op'DDD, the islet cell toxicity of streptozotocin and the abortifacient action of methotrexate. In this wide variety of drugs and tumours, the determinant of antitumour response is how closely the tumour resembles the normal tissue. In the case of the corticoids, there is good evidence that the lympholytic action follows the binding of the corticoid in the cytoplasm by a protein mechanism resembling that for the sex steroids and progestin. However, the subsequent events do not seem to involve proliferation or differentiation into a stable end-cell; rather they result in death of the lymphocyte. This suggests that the ultimate differentiated fate of the lymphocyte is to lyse and deliver its products to the rest of the body. The possibility that comparable binding proteins might be involved in the action of Op'DDD and streptozotocin needs careful investigation.

In the case of l-asparaginase, a remarkable logical error was committed by some who failed to recognize the primary lympholytic effects of the drug and erroneously attributed its action to a " unique requirement " by tumour for asparagine. We now realize that precisely the opposite is the case, that asparaginase works only on lymphatic tumour cells which are very like normal lymphocytes in their asparagine dependency. A number of predictive tests have been devised for this agent:(1) The amount of asparaginase synthetase in a tissue can be used to determine freedom from asparagine dependency and hence from asparaginase effect; (2) Cells made resistant to l-asparaginase *in vitro* also change their membranes to contain fewer asparagine residues, since these residues in drug sensitive cells appear to be hydrolysed, with rupture of the cell (Kessel and Bosman, 1972). Since asparagine forms one of the major links between the protein backbone and sugar residues in the cell membrane, the reduction of asparagine residues in l-asparaginase resistance is also accompanied by a decrease in total anthrone positive material in the membranes of such resistant cells in culture. This forms the basis of a predictive test for asparagine sensitivity and an explanation for the resistance of myelogenous cells to asparaginase which is currently under study in our laboratories.

*Mitotoxic* chemotherapy is commonly, but erroneously, thought of as the only type of chemotherapy. Such therapy, directed against the proliferative qualities of tumours, carries an unavoidable burden of toxicity to the rapidly growing tissues of the host, particularly the gastrointestinal epithelia, bone marrow and skin with its appendages. The antimetabolites comprise a major subclass of such agents, with methotrexate being the type compound for folate antagonists designed to inhibit dihydrofolate reductase (dHFR) activity and in so doing prevent the biosynthesis of *de novo* thymidine. Although methotrexate induced resistance is accompanied by increases in intracellular content of dHFR in some animal tumours, Roberts and Hall (1969) found that the " innate " sensitivity of mouse tumours which had not been exposed to the drug was not related to dHFR concentration. Kessel, Hall and Roberts (1968) found that the actual *in vivo* determinant of innate sensitivity was transport of the drug into mouse and human leukaemic cells. This sequence of experiments pointed out that: (1) there were great differences between " innate " and drug-induced or " acquired " drug resistance, to say nothing of " collateral " changes in sensitivity to one drug acquired *pari-passu* during treatment with a quite different drug; (2) " innate " drug sensitivity may commonly be explained by a single predictive determinant, but in cases of drug induced resistance, more than one mechanism is commonly involved, as for example, a drop in transmembrane transport of methotrexate, plus an elevation of dHFR, plus a switch from the utilization of deoxyuridylate to thymidylate for DNA synthesis.

Six-mercaptopurine (6 MP), like most antimetabolite bases, diffuses freely into target cells but requires phosphorylation to a nucleotide. A predictive test for sensitivity to 6 MP can thus be designed for rodent and for human leukaemic cells, based upon the activity of a guanine-hypoxanthine phosphoribosyl-transferase. This assay does not predict well unless whole cells are used, and innate sensitivity is being sought (Kessel and Hall, 1969). In certain leukaemias which were treated with 6 MP, the development of acquired resistance could be shown also to be correlated with the appearance of a phosphatase which hydrolysed the 6 MP nucleotide, so that separate tests may be needed for clinical use depending upon the prior exposure of the patient to the drug (Wollpert *et al.*, 1971). It would be of great interest to check whether the virtually complete resistance of human solid tumours to 6 MP was due to a relative absence of guanine-hypoxanthine phosphoribosyltransferase from such tumours.

In the case of 5-fluorouracil, conversion to a nucleotide is necessary for the activation of the drug, and anti-tumour activity is proportional to the amount of FUMP formed. In human leucocytes the lack of both nucleoside phosphorylase, to convert FU to FUR, and of a pyrimidine phosphoribosyltransferase, to convert FU to FUMP, makes human leukaemic cells resistant to FU (Hall *et al.*, 1968). It has been estimated that the application of these predictive tests at a cost of possibly $10,000 could have prevented clinical trials of FU and FUdR in human leukaemia costing 20 times as much, to say nothing of the loss of critical treatment time by the patients involved. In the case of animal solid tumours, the determining enzyme appears to be the pyrimidine phosphoribosyltransferase, and preliminary studies to date suggest that the same enzyme is predictive for response of colon and breast cancers to 5-fluorouracil (Keyes and Hall, 1969).

Phosphorylation is also the determinant of activity of nucleosides such as cytarabine, both in murine and human leukaemic cells (Kessel, Hall and Rosenthal, 1969). For a while, deamination of cytarabine by target cells was thought to be a possible determinant of response, but it does not appear that this enzyme plays an important role in the prediction of response (Stewart and Burke, 1971; Hall and Levine, 1967). The relative ineffectiveness of cytarabine in the treatment of human solid tumours suggests that there is a relative lack of deoxycytidine kinase in such tumours.

The alkylating agents and radiation both produce nicks and defects in the DNA molecule and there is evidence, from the shape of the shoulder at low doses on the survival curves for both agents, that DNA repair may be involved. This is also suggested by animal work on myeloma with repair inhibitors and by the increased radiation sensitivity of cells such as those in xeroderma pigmentosum, in which repair is defective and delayed (Setlow *et al.*, 1969). We are at present developing an assay for potential radiation and alkylating agent sensitivity based upon the DNA repair capacity of the cells to be treated (Leiberman *et al.*, 1971).

Many of the larger heterocyclic molecules seem to have difficulty in getting transported across the cell membranes of animal tumour cells which are resistant to the drugs in question (Kessel and Bosmann, 1970). Induction of resistance to actinomycin D, and to vincristine, daunorubicin and some terephthalanalides, can be shown to be mediated by a similar mechanism in which there is more transfer of glycoprotein residues into the tumour cell membrane and an accumulation of anthrone-positive material, suggesting a thickened cell membrane (Bosmann and Kessel, 1971). It is interesting to speculate whether these characteristics are more like those usually found in the glycocalyx of an epithelial cell, even a tumour cell, as contrasted with the thinner cell membranes of mesenchymal cells; possibly such differences could permit easier ingress of such molecules to the mesenchymal tumour cells of sarcomata and lymphomata and thus predict the relatively greater effectiveness of such compounds for mesenchymal as opposed to epithelial tumours. We were able to predict camptothecin resistance by rendering a murine lymphoma resistant to actinomycin D and daunorubicin. These concepts contain the seeds of a prediction system for this whole class of compounds which we are presently studying. If such membrane changes result in similar lack of uptake by human tumours, it might be simpler to give a pulse dose of the compound in question in tracer labelled form and measure the differential uptake observed at subsequent biopsy.

When the uptake of methotrexate or of actinomycin and similar compounds is at an intermediate level, or the amount of antimetabolite nucleotide accumulated intracellularly is at an equivocal level, it may be possible to increase the efficiency of the prediction system by giving a test dose of the drug in question and following the subsequent effects on macromolecular biosynthesis (Hall *et al.*, 1973). This can only be done in tumours which permit repeated biopsies, such as in a patient with multiple skin masses, or in leukaemia, or in the case of malignant effusions. The uptake of $^{32}P$ or an $^{131}I$ or $^{125}I$ labelled base or nucleoside can also be measured by external radioisotopic monitors, but the data tend to be less precise because of the problems of collimation and quantitation (Nathanson, 1971).

In the other instances, single ineffective drugs to not cause a drop in the 24-hour utilization of UdR (deoxyuridine) or TdR (thymidine) for DNA synthesis, and such drugs can therefore be omitted from the treatment programme. After one effective drug is found, the DNA synthesis may not return to near the initial rate for some time, and thus impair the testing of subsequent drugs.

### APPLICATION

The application of such predictive tests has resulted in improved selection of drug regimens for the treatment of acute myelogenous leukaemia. The optimal combination of tests included measurements of uptake and phosphorylation of methotrexate, 6 MP and cytarabine, followed by observations on serial changes in DNA synthesis following administration of single full-dose pulses when uptake levels were intermediate and of cyclophosphamide, vincristine and daunorubicin. With this method, there were no false negative or positive results. The number of responses was not altered but the failures were identified and could have had their treatment stopped in favour of newer and more promising agents.

In the case of breast cancer, oestrogen receptors have been shown to be predictive of response to oophorectomy, adrenalectomy and hypophysectomy (oncoprival therapy). The data on the ontoductive effects of massive pharmacological doses of the sex steroids on breast cancer are not numerous yet, but all those available from a number of sources are consistent in support of a predictive role in ontoductive therapy for tumour steroid receptors.

The data are accumulating on colon and breast cancer with regard to treatment with 5-fluorouracil, and at the present time suggest that low levels of pyrimidine phosphoribosyltransferase do not permit response to FU, whereas high levels in the tumours predispose to 5 FU induction of response.

Further studies are under way in respect of DNA repair in human lymphoma response to mustards, and of the multiple factors involved in predicting response to lymphoma and breast treatment combinations.

### SUMMARY

*In vitro* measurements of drug–tumour interactions have not been of help in selecting improved therapeutic regimens for individual patients. Studies of human pharmacokinetics on cell kinetics have also not been of help. *In vivo* and *in vitro* measurements of drug handling by and drug effects upon tumour tissue, however, have been of considerable help.

At present a *definite* test for methotrexate, 6-mercaptopurine, 6-thioguanine and cytarabine sensitivity in leukaemias is the uptake and retention of the active form of the drug. The presence of an oestrogen receptor appears to predict well for response to the oncoprival, ontoductive and ontotoxic effects of steroids.

A number of other tests *appear* to predict well for response to therapy. These include breast and colon tumour levels of the phosphoribosyl-transerase for 5-fluorouracil, and the reduction and binding of testosterone by breast tissue. Differential tumour uptake of actinomycin D, adriamycin and mustards are also in this category. Receptors for corticoids and progestins appear ready to exploit in the treatment of lymphoproliferative cancers and endometrial cancer.

Tests which *show promise* of applications to the human situation include the measurements of membrane glycosidases and glycoproteins transferases for heterocyclic compounds, and the measurement of specific repair enzymes for sensitivity to the alkylating agents and to x-irradiation.

The original work referred to is the product of collaborative efforts with Drs David Kessel, H. Bruce Busmann, Aly Nahas, De Wayne Roberts and Bruce Hacker.

REFERENCES

BOSMANN, H. B. & KESSEL, D. (1967) Altered Glycosidase Levels in Drug Resistant Mouse Leukaemias. *Molec. Pharm.*, **6**, 345.

HALL, T. C. & LEVINE, R. (1967) Deamination of Cytosine Arabinoside by Normal and Malignant Tissues. *Proc. Am. Ass. Cancer Res.*, **8**, 24.

HALL, T. C., KESSEL, D., GODSILL, A. & ROBERTS, D. (1968) Uridine Phosphorylation, an Overlooked Pathway?; 5-fluorouridine, a Neglected Drug? *Proc. Am. Ass. Cancer Res.*, **9**, 27.

KESSELL, D., HALL, T. C. & ROBERTS, D. (1968) Modes of Uptake of Methotrexate by Normal and Leukemic Human Leukocytes, *in vitro* and Their Relation to Drug Response. *Cancer Res.*, **28**, 564.

KESSEL, D. & HALL, T. C. (1969) Retention of 6-Mercaptopurine by Intact Cells as an Index of Drug Responsiveness in Human and Murine Leukemias. *Cancer Res.*, **29**, 2116.

HALL, T. C., KESSEL, D., OKADA, K., HACKER, B., LECHNER, D. & ROBERTS, D. (1973) Patterns of Drug Effects on Nucleic Acid Synthesis in Human Leukemia. Chapter in: Erythrocytes, Thrombocytes, Leucotyes. In *Recent Advances in Membrane and Metabolic Research*. Eds. Gerlach, Moser, Deutsch and Willmanns. Stuttgart: Geo. Thieme Verlag.

KESSEL, D., HALL, T. C. & ROSENTHAL, D. S. (1969) Uptake and Phosphorylation of Cytosine Arabinoside by Normal and Leukemic Blood Cells. *Cancer Res.*, **29**, 459.

KESSEL, D. & BOSMANN, H. B. (1970) On the Characteristics of Actinomycin D Resistance in L5178 Cells. *Cancer Res.* **30**, 2695.

KESSEL, D. & BOSMANN, H. B. (1972) L-asparaginase Effects on Intact Murine Leukemic Cells and Isolated Cell Plasma Membranes. *Biochim. biophys. Res. Commun.* **48**, 35.

LIEBERMAN, M. W., BANEY, R. N., LEE, R. E., SELL, S. & FARBER, E. (1971) Studies on DNA Repair in Human Lymphocytes treated with Proximate Carcinogens and Alkylating Agents. *Cancer Res.*, **31**, 1297.

NATHANSON, L. (1971) *In Vivo* Prediction of Drug Sensitivity with Cancer-seeking Isotopes. Chapter in NCI Monog. No. 34 *Prediction of Response to Cancer Therapy*. Ed. T. C. Hall. Washington: U.S. Govt Printing Office.

REYES, P. & HALL, T. C. (1969) Synthesis of 5-fluorouridine 5' phosphate by a Pyrimidine Phosphoribosyltransferase of Mammalian Origin. II Correlation between Tumor Levels of the Enzyme and the 5-fluorouracil Promoted Increase in Survival of Tumor-bearing Mice. *Biochem. Pharmac.*, **18**, 2587.

ROBERTS, D. & HALL, T. C. (1969) Enzyme Activities and Deoxynucleoside Utilisation by Leukemic Leukocytes in Relation to Drug Therapy and Resistance, *Cancer Res.*, **29**, 166.

SETLOW, R. B., REGAN, J. D., GERMAN, J. & CARRIER, W. L. (1969) Evidence that Xeroderma Pigmentosum Cells do not Perform the First Step in the Repair of Ultraviolet Damage to their DNA, *Proc. natn. Acad Sci. U.S.A.*, **64**, 1035.

STEWAPT, C. D. & BURKE, P. J. (1971) Cytidine Deaminase and the Development of Resistance to Arabinosyl Cytosine. *Nature, New Biol.*, **223**, 109.

WOLLPERT, K. C. ET AL. (1971) Mechanisms of Resistance to 6-Thiopurines; Role of Phosphohydrolases. *Cancer Res.*, **31**, 1620.